# Assessment of reproductive toxicity of gold nanoparticles and its reversibility in male albino rats

**DOI:** 10.1007/s43188-023-00203-2

**Published:** 2023-08-23

**Authors:** Nancy A. Abdulhaq, Dina A. Elnady, Hend M. Abo El-atta, Doaa A. El-Morsi, Seham A. Gad El-Hak

**Affiliations:** 1 Forensic Medicine and Clinical Toxicology Department, Faculty of Medicine, Mansoura University, Mansoura, Egypt; 2 Pathology Department, Faculty of Medicine, Mansoura University, Mansoura, Egypt; 3 Medical Education Department, Faculty of Medicine, Mansoura University, Mansoura, Egypt; 4 Medical Education Department, Faculty of Medicine, Delta University for Science and Technology, Belqas, Egypt

**Keywords:** Gold nanoparticles, Reproductive toxicity, Spermatogenesis, Withdrawal, Reversibility

## Abstract

Nanotechnology has become a trending area in science all over the world. Although gold nanoparticles (AuNPs) have been utilized widely in biomedical fields, potential toxicities may arise from their interactions with biological systems. The current study aimed at evaluating the toxic effects of AuNPs on the reproductive system of adult male albino rats and assessing the recovery probability. In this study, AuNPs (13 ± 4 nm in diameter) were synthesized, and the experimental work was conducted on 60 adult male albino rats divided into the following groups: control group (received deionized water daily intraperitoneally (IP) for 28 days), test group, and withdrawal groups I and II (received 570 μg/kg of 13 ± 4 nm AuNPs daily IP for 28 days). Withdrawal groups I and II were left for another 30 and 60 days without sacrification, respectively. The test group showed significant decreases in final body and absolute testicular weights, testosterone hormone level, sperm count and motility, and spermatogenesis score, as well as significant increase in the percentage of sperms of abnormal morphology compared to the control group, associated with significant light and electron microscopic histopathological changes. Partial improvement of all studied reproductive parameters was detected after one month of withdrawal in withdrawal group I, and significant improvement and reversibility of all these parameters were reported after two months of withdrawal in withdrawal group II. So, AuNPs induce male reproductive toxicity, which partially improves after one month of withdrawal and significantly improves and reverses after two months of withdrawal.

**Supplementary Information:**

The online version contains supplementary material available at 10.1007/s43188-023-00203-2.

## Introduction

Nanotechnology has attracted the wondrous interest of researchers and scientists globally and that addresses the development of functional materials and devices with a dimensional range of 1–100 nm. Among various classes of these nanomaterials, gold nanoparticles (AuNPs) are highly remarkable due to their unique properties that offer a wide range of biomedical and industrial applications, including cosmetics, chemical sensing, drug carriers, bioimaging, cancer treatment, and gene therapy [10].

Despite the huge potential benefits of AuNPs in the areas of biomedical and industrial applications and their reported safety attributed to their inert and non-toxic gold core, there has been an increased interest in studying their possible deleterious effects on biological systems due to their ability to induce oxidative stress. Unfortunately, the results of these studies did not yield univocal reports [22].

Reproductive toxicological studies are mandatory at every stage of the drug approval process. These studies are of fundamental importance as possible defects may not only affect the person or animal directly treated with the drug but also have possible adverse effects on the following generations [8]. Exposure to nanoparticles (NPs) may induce different deleterious effects on male reproductive organs, spermatogenesis and hormone levels [16].

Previous studies reported that AuNPs exposure led to a decrease in sperm motility, morphology, and fertilizing capability, an increase in the number of abnormal spermatozoa [28, 32, 36], an accumulation of AuNPs in the testes [35], a reduction in population of germ cells, degeneration of testicular tissues, detachment of germinal epithelium from the basement membrane [10], and a reduction of testosterone production [21]. Unfortunately, the reversibility of AuNP-induced reproductive toxicity was not investigated in those studies.

The aim of this work is to evaluate the toxic effect of AuNPs on the reproductive system of adult male Albino rats and to explore the reversibility of AuNP-induced reproductive toxicity after one and two months of withdrawal.

## Materials and methods

The current experimental study was conducted on 60 adult male Albino rats weighing between 150 and 200 g purchased from Mansoura Experimental Research Centrer (MERC), Faculty of Medicine, Mansoura University. Rats were housed in clean cages, kept under standard laboratory conditions including good lighting (12 h of light and 12 h of darkness) and good aeration, and fed a standard laboratory diet and tap water ad libitum for 14 days before and during the experiment. The experimental protocol was approved by Mansoura University Institution Research Board (IRB) (Code Number: MD.19.01.124).

### Material

#### Chemicals and kits

Gold (III) chloride hydrate purchased from Sigma-Aldrich, USA; trisodium citrate purchased from EL Gomhorya Company, Egypt; deionized water; and a kit for the analysis of serum testosterone purchased from EQUIPAR Diagnostic, Serono, Italy, were used in the current study.

### Methods

#### Gold nanoparticles preparation and characterization

**Fig. 1:**
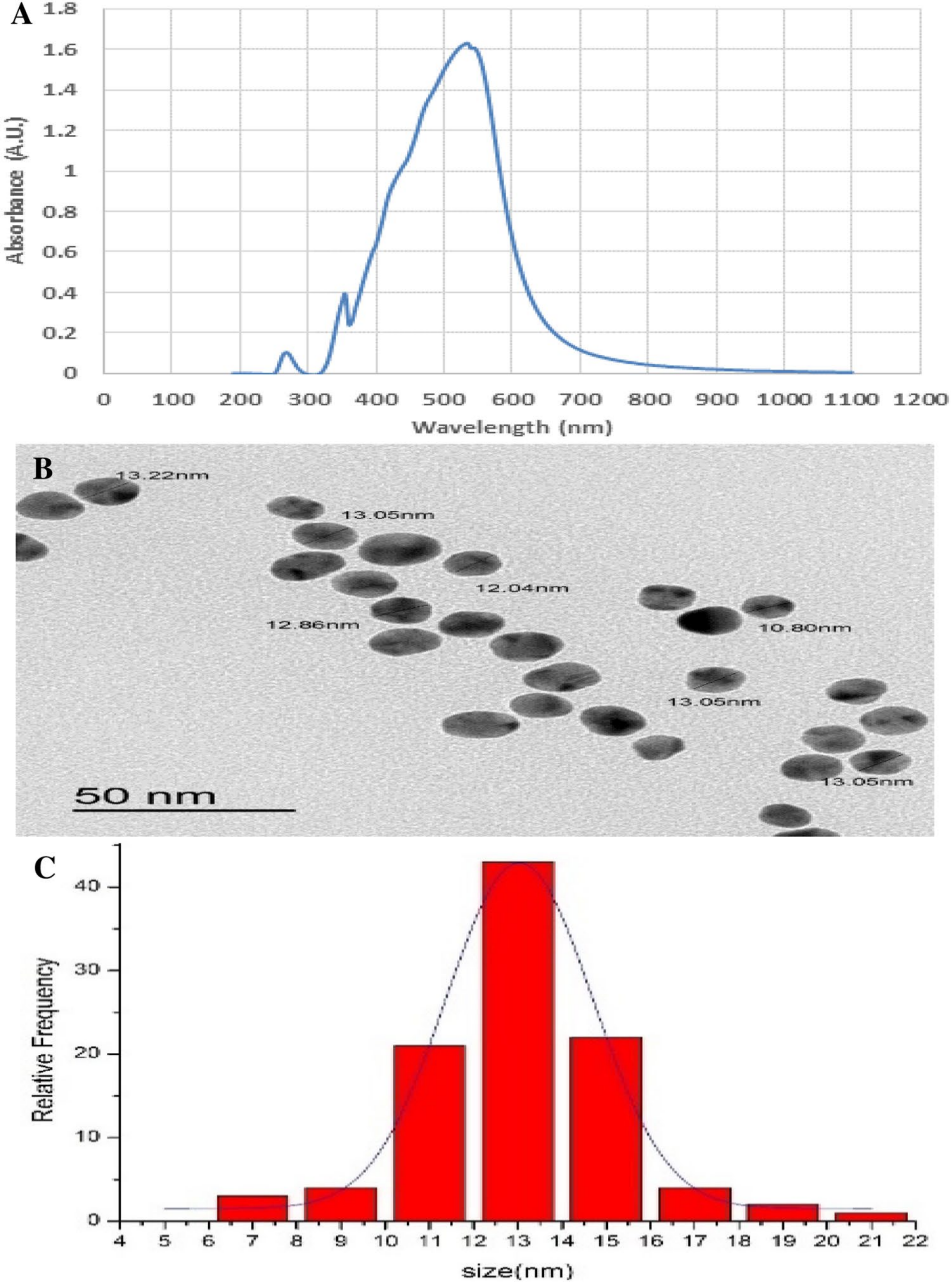
Characterization of gold nanoparticles. **a** Ultraviolet–visible spectrum of gold nanoparticles showing sharp absorbance band peaked at wavelength 520 nm. **b** High resolution transmission electron microscope image displaying spherical shape of AuNPs with average size 13 ± 4 nm, scale bar was 50 nm. **c** Histogram representing size distribution of AuNPs analyzed using ImageJ

Preparation and characterization were performed at Faculty of Science, Mansoura University. Colloidal AuNPs were prepared according to the standard citrate reduction method [24]. Then, they were characterized using ultraviolet–visible absorption spectroscopy, transmission electron microscope (TEM), size distribution histogram, and atomic absorption spectrophotometer with the following criteria: dark wine-red colored solution, spherical in shape with an average size 13 ± 4 nm, and 384 mg/L in concentration (Fig. 1).

#### Study design

Rats were weighted and divided randomly into four groups (15 rats each) as follows:

- Control group: rats received deionized water daily IP for 28 days.
- Test group: rats received a toxic dose of AuNPs (570 μg/kg) daily IP for 28 days [35].
- Withdrawal group I: rats received a toxic dose of AuNPs (570 μg/kg) daily IP for 28 days. Then, they were left with free access to tap water and food to assess the recovery for another 30 days.
- Withdrawal group II: rats received a toxic dose of AuNPs (570 μg/kg) daily IP for 28 days. Then, they were left with free access to tap water and food to assess the recovery for another 60 days.

#### Sample collection and preparation

After twenty-four hours from the last dose in both control and test groups and 30 and 60 days from the last dose in both withdrawal groups I and II, respectively, rats were weighted and anaesthetized IP with sodium pentobarbital (40 mg/kg) [40]. From each rat, one ml of blood was obtained from the apex of the heart for determination of testosterone hormone level. The epididymides and testes were dissected out. The epididymis was sharply cut and separated from the testis. Each epididymis was gently squeezed on a slide and then pulled up using the leukocyte pipette of the hemocytometer for calculation of its volume. Then, the viscid epididymal spermatozoan fluid was allowed to liquefy with 0.5 ml of hydroxyethyl piperazineethanesulfonic acid buffered Earle's balanced salt solution and 0.4% human albumin solution for semen analysis as regards count, motility, and morphology [9].

The right testes were weighted in grams to evaluate absolute testicular weights. Then, they were prepared and stained with hematoxylin and eosin (H & E) stain and Masson's trichrome stain for light microscopic histological examination and digital morphometric study. On the other hand, the left testes were prepared and stained with 1% toluidine blue for electron microscopic examination by TEM.

#### Light microscopic study

In this study, spermatogenesis and histopathological testicular changes were evaluated. A semi-quantitative evaluation of spermatogenesis was performed using Johnsen’s tubular biopsy score (JTBS) of spermatogenesis in 20 seminiferous tubules from each testicular section. As described by Johnsen, testicular tubular sections in each group were evaluated and given a score from 1 to 10, as shown in Table 1. JTBS was calculated by dividing the sum of all scores by the total number of seminiferous tubules examined [39].

**Table 1:** Johnsen’s tubular biopsy score according to histologic criteria of spermatogenic cells

Score	Histologic criteria of spermatogenic cells
10	Complete spermatogenesis, numerous spermatozoa, germinal epithelium of regular height and tubular lumen of normal diameter
9	Numerous spermatozoa, germinal epithelium disorganized with sequestration of germinal cells and tubular lumen obturated
8	Less than 5 ± 10 spermatozoa per tubular cross-section
7	No spermatozoa, but numerous spermatids, spermatocytes and spermatogonia
6	No spermatozoa, 5 ± 20 spermatids and numerous spermatocytes and spermatogonia per cross-section
5	No spermatozoa and spermatids, but numerous spermatocytes and spermatogonia
4	No spermatozoa and spermatids, less than 5 spermatocytes, but numerous spermatogonia per cross-section
3	Only spermatogonia
2	No germinal cells, only Sertoli cells (Sertoli-cell-only syndrome)
1	No cells at all within the tubules

In addition, a semi-quantitative evaluation of histopathological testicular changes was performed as follows: Testicular sections were examined and scored for the histopathological changes [0 (no injury), 1 (mild injury), 2 (moderate injury), 3 (severe injury)]; thirty seminiferous tubules from each rat were examined randomly; each tubule took a score of (0, 1, 2 or 3); the histopathological changes were categorized into seven parameters: (a) disruption of seminiferous tubules, (b) detachment of spermatogenic cells, (c) inflammation, (d) *edema* of the interstitium, (e) congestion of vessels, (f) degeneration of Sertoli cells, and (g) degeneration of Leydig cells; for each experimental group, the average scores for slides were taken and assessed statistically [18].

Moreover, Masson's trichrome-stained slides were photographed using an Olympus® digital camera installed on an Olympus® microscope with a 0.5 X photo adaptor and a 10 or 20 X objective. The resulting images were analyzed on an Intel® Core I7®-based computer using Video Test Morphology® software (Russia) for calibrated distance measurement, area measurement, and descriptive geometric analysis. The digital morphometric study of the thicknesses of tunica albuginea, blood vessel walls, and epithelial lining of the tubules, as well as their mean diameter, was performed as follows: One image was tested for each animal in each group for each parameter; in each image, six measurements for the parameter were taken at different random places using the manual line tool; all measurements were calibrated against a micrometer slide, which was photographed using the same optical system. This process enables the system to measure the distances in (μm) instead of pixels. Then, these measurements were averaged for each image [1].

#### TEM Study

TEM study was performed at the Electron Microscopy Unit, Faculty of Science, Alexandria University.

### Statistical analysis

The collected data were coded, processed, and analyzed using the computerized Statistical Package for Social Science (SPSS) program (version 22.0). Student t-tests, one-way ANOVA tests, post-hoc Tukey tests, Kruskal–Wallis tests, and Mann–Whitney *U* tests were performed for statistical comparisons. Descriptive statistics were performed only for TEM histopathological images. Qualitative data were described using numbers and percents. While quantitative data were described using the median for non-parametric data and the mean ± standard deviation (SD) for parametric data after testing normality using the Shapiro–Wilk test, The significance of the obtained results was judged when the *p* value was ≤ 0.05.

## Results

### Body weight changes in animals

After receiving the toxic dose of AuNPs for 28 days, there was a statistically significant decrease in final body weights in animals of the test group compared to animals of the control group (*p1* = 0.048). Meanwhile, the final body weights of withdrawal group II animals showed a statistically significant increase compared to the test group and withdrawal group I (*p5* = 0.001 and *p6* = 0.01, respectively) (Table 2).

**Table 2:** Comparisons between initial and final body weights (g) and percentage of weight change among all studied groups (n = 60)

Groups (n = 15 for each)	Initial body weight (g) Mean ± SD	Final body weight (g) Mean ± SD	Comparison between initial and final body weights	Percentage of weight change
Control group	166.5 ± 9.44	210.6 ± 32.5	< 0.001*	26.5%
Test group	170.70 ± 16.45	181 ± 17.99	0.087	9.0%
Withdrawal group I	158.6 ± 10.58	197.1 ± 4.80	0.024*	24.3%
Withdrawal group II	171.40 ± 15.03	236.1 ± 24.71	< 0.001*	37.7%
Comparison of final body weights among all studied groups	Test of significance		Paired comparison	
	F = 5.2 *p* = 0.004*		*p1* = 0.048*	
			*p2* = 0.357	
			*p3* = 0.086	
			*p4* = 0.273	
			*p5* = 0.001*	
			*p6* = 0.01*	

*n* number, *g* gram, *SD* standard deviation, *p* comparison among all studied groups, *p1* comparison between control group and test group, *p2* comparison between control group and withdrawal group I, *p3* comparison between control group and withdrawal group II, *p4* comparison between test group and withdrawal group I, *p5* comparison between test group and withdrawal group II, *p6* comparison between withdrawal groups I and II, *F* One Way ANOVA test, *p is significant

### Absolute testicular weights of animals

As regards absolute testicular weights, the test group showed a statistically significant decrease compared to the control group (*p1* = 0.002). However, withdrawal group II showed a statistically significant increase compared to the test group and withdrawal group I (*p5* = 0.001 and *p6* = 0.004, respectively) (Table 3).

**Table 3:** Comparisons of absolute testicular weights (g) among all studied groups of rats (n = 60)

Groups (n = 15 for each)	Absolute testicular weights (g) Mean ± SD	Test of significance
Control group	1.37 ± 0.15	F = 7.32
		*p* = 0.001*
Test group	1.08 ± 0.21	Paired comparison
Withdrawal group I	1.15 ± 0.13	*p1* = 0.002*
		*p2* = 0.014*
		*p3* = 0.635
		*p4* = 0.408
		*p5* = 0.001*
		*p6* = 0.004*
Withdrawal group II	1.40 ± *0.2*4	

*n* number, *g* gram, *SD* standard deviation, *p* comparison among all studied groups, *p1* comparison between control group and test group, *p2* comparison between control group and withdrawal group I, *p3* comparison between control group and withdrawal group II, *p4* comparison between test group and withdrawal group I, *p5* comparison between test group and withdrawal group II, *p6* comparison between withdrawal groups I and II, *F* One Way ANOVA test, *p is significant

### Serum testosterone hormone levels

The test group showed a statistically significant decrease in serum testosterone hormone levels compared to the control group (*p1* < 0.001). While, withdrawal groups I and II showed statistically significant increases compared to the test group (*p4* and *p5* < 0.001) (Table 4).

**Table 4:** Comparison of serum testosterone hormone levels (ng/dL) among all studied groups of rats (n = 60)

Groups (n = 15 for each)	Serum testosterone hormone levels (ng/dL) Mean ± SD	Test of significance
Control group	190.06 ± 30.58	F = 49.69
		*p* < 0.001*
Test group	84.79 ± 20.74	Paired comparison
Withdrawal group I	161.40 ± 20.91	*p1* < 0.001*
		*p2* = 0.01*
		*p3* = 0.20
		*p4* < 0.001*
Withdrawal group II	203.97 ± 21.84	*p5* < 0.001*
		*p6* < 0.001*

*n* number, *ng* nanogram, *dL* deciliter, *SD* standard deviation, *p*: comparison among all studied groups, *p1* comparison between control group and test group, *p2* comparison between control group and withdrawal group I, *p3* comparison between control group and withdrawal group II, *p4* comparison between test group and withdrawal group I, *p5* comparison between test group and withdrawal group II, *p6* comparison between withdrawal groups I and II, *F* One Way ANOVA test, *p is significant

### Semen analysis

By comparing semen analysis parameters (sperm count, motility, and abnormal morphology) among all studied groups, the test group showed statistically significant decreases in sperm count and percentage of motile sperms and a statistically significant increase in the percentage of sperms with abnormal morphology compared to the control group (*p1* < 0.001 for all) (Table 5).

**Table 5:** Comparison of semen analysis parameters (sperm count, motility and abnormal morphology) among all studied groups of rats (n = 60)

Groups (n = 15 for each)	Sperm count (10^6^/ mL) Mean ± SD	Sperm motility (%) Mean ± SD	Abnormal sperm morphology (%) Median (min—max)
Control group	111.68 ± 9.06	93.0 ± 2.58	4 (0—6)
Test group	56.54 ± 12.97	52.50 ± 6.34	20 (10—25)
Withdrawal group I	76.52 ± 14.72	77.5 ± 9.20	10 (5—15)
Withdrawal group II	112.26 ± 17.26	85.50 ± 8.32	5 (0—10)
Test of significance	F = 39.47	F = 61.66	KW
	*p* < 0.001*	*p* < 0.001*	*p* < 0.001*
Paired comparison	*p1* < 0.001*	*p1* < 0.001*	*p1* < 0.001*
	*p2* < 0.001*	*p2* < 0.001*	*p2* < 0.001*
	*p3* = 0.926	*p3* = 0.069	*p3* = 0.284
	*p4* = 0.003*	*p4* < 0.001*	*p4* < 0.001*
	*p5* < 0.001*	*p5* < 0.001*	*p5* < 0.001*
	*p6* < 0.001*	*p6* = 0.002*	*p6* = 0.008*

*n* number, *mL* milliliter, *SD* standard deviation, *min* minimum, *max* maximum. *p* comparison among all studied groups. *p1* comparison between control group and test group. *p2* comparison between control group and withdrawal group I, *p3* comparison between control group and withdrawal group II. *p4* comparison between test group and withdrawal group I. *p5* comparison between test group and withdrawal group II, *p6* comparison between withdrawal groups I and II. *F* One Way ANOVA test, *KW* Kruskal Wallis test, *p is significant

On the other hand, withdrawal group I showed a statistically significant increase in sperm count and percentage of motile sperms associated with a statistically significant decrease in the percentage of sperms with abnormal morphology compared to the test group (*p4* = 0.003, < 0.001 and < 0.001 respectively).

Moreover, withdrawal group II showed statistically significant increases in sperm count and percentage of motile sperms associated with a statistically significant decrease in the percentage of sperms with abnormal morphology compared to the test group (*p5* < 0.001 for all) and withdrawal group I (*p6* < 0.001 for all).

### Light microscopic results

#### Johnsen's tubular biopsy score for spermatogenesis (JTBS)

Spermatogenesis was compared among all studied groups of rats by comparing JTBS, as shown in Table 6. The test group shows a statistically significant decrease in JTBS compared to the control group (*p1* < 0.001). While, withdrawal groups I and II showed statistically significant increases in JTBS compared to the test group (*p4* and *p5* < 0.001).

**Table 6:** Comparison of Johnsen's tubular biopsy scores for spermatogenesis among all studied groups of rats (*n* = 60)

Groups (n = 15 for each)	Johnsen's tubular biopsy score for spermatogenesis (ng/dL) Mean ± SD	Test of significance
Control group	9.46 ± 0.11	F = 31.41
		*p* < 0.001*
Test group	3.76 ± 2.69	Paired comparison
Withdrawal group I	7.74 ± 1.10	*p1* < 0.001*
		*p2* = 0.014*
		*p3* = 0.675
Withdrawal group II	9.18 ± 0.59	*p4* < 0.001*
		*p5* < 0.001*
		*p6* = 0.037*

*n* number, *SD* standard deviation, *p* comparison among all studied groups, *p1* comparison between control group and test group, *p2* comparison between control group and withdrawal group I, *p3* comparison between control group and withdrawal group II, *p4* comparison between test group and withdrawal group I, *p5* comparison between test group and withdrawal group II, *p6* comparison between withdrawal groups I and II, *F* One Way ANOVA test, *p is significant

#### Testicular histopathological changes

**Fig. 2:**
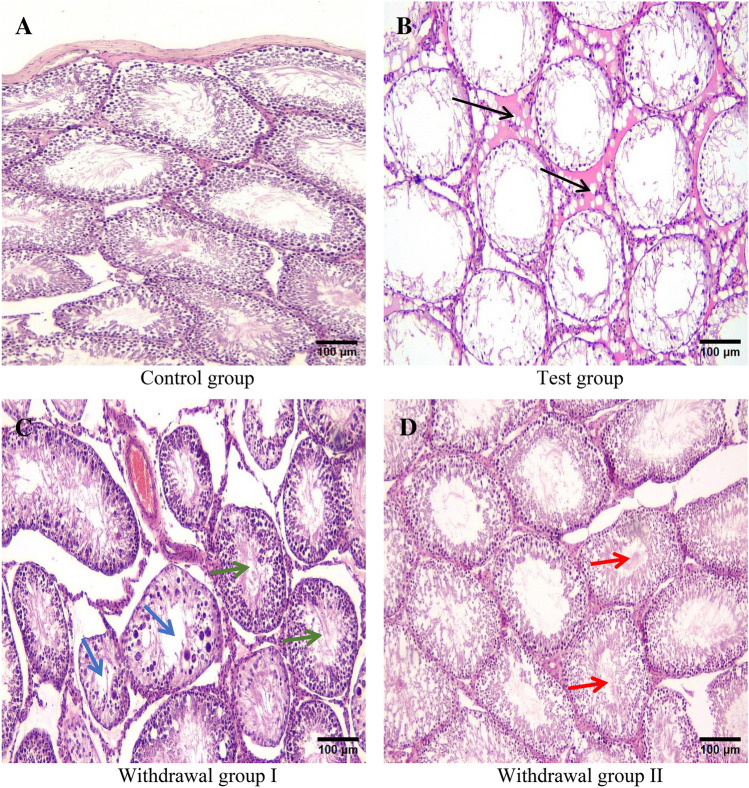
Photomicrographs of paraffin sections in the testes of the control group (**a**) showing normal testicular architecture; the test group (**b**) showing vacuolated tubules separated by interstitial exudate and inflammatory cells (black arrows); withdrawal group I (**c**) showing preservation of the germinal epithelium in some tubules (green arrows), degeneration and vacuolation in other tubules (blue arrows); withdrawal group II (**d**) showing layers of spermatogenic cells lining the tubules, mature spermatozoa in the lumen (red arrows) (H & E stain X100). Scale bar: 100 μm

**Fig. 3:**
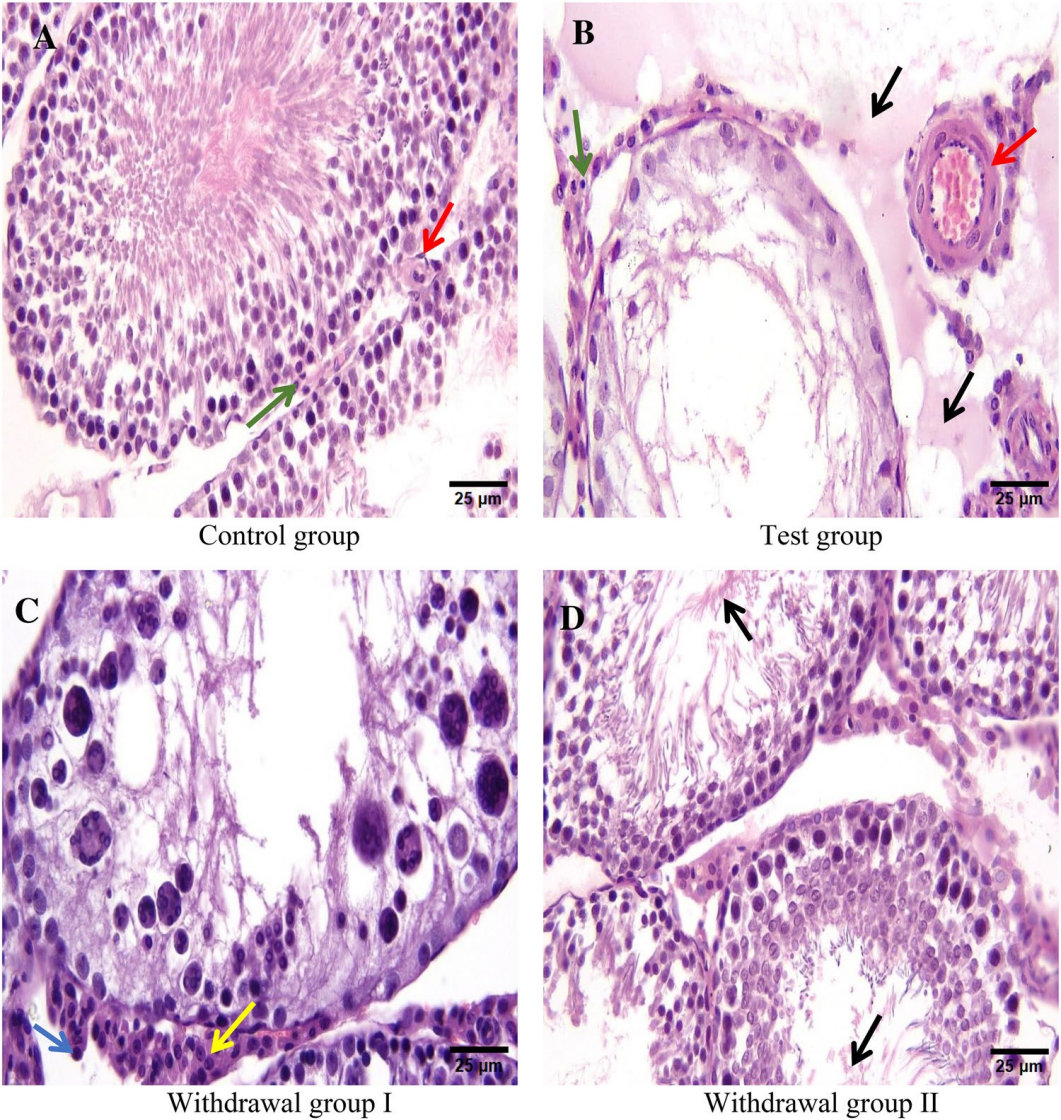
Photomicrographs of paraffin sections in the testes of the control group (**a**) showing seminiferous tubules lined by layers of spermatogenic cells separated by interstitium containing blood vessels (red arrow) and normal Leydig cells (green arrow); the test group (**b**) showing marked reduction of stratified germinal epithelium in the tubules, most of spermatogenic cells are replaced by vacuoles, the lumen of the tubule is devoid of mature spermatozoa, tubules are lined by degenerated Sertoli cells and separated by interstitium containing thickened blood vessels (red arrow), degenerated Leydig cell (green arrow), exudate and inflammatory cells (black arrows); withdrawal group I (**c**) showing tubules lined by degenerated cells, no mature spermatozoa are present in the lumen of seminiferous tubules, some Leydig cells are normal with rounded to oval nuclei and prominent nucleoli (yellow arrow), others are degenerated with pyknotic nuclei (blue arrow); withdrawal group II (**d**) showing preservation of germinal epithelium in most of tubules, mature spermatozoa are seen in the lumen (black arrows) (H & E stain X400). Scale bar: 25 μm

Light microscopic examination of testicular tissue sections from control group rats revealed normal testicular architecture. The testis was surrounded by tunica albuginea, which was formed of connective tissue fibers and fibroblasts (Fig. 2a). The testis consisted of seminiferous tubules of variable sizes and shapes separated by interstitial tissue containing small blood vessels and groups of Leydig cells close to them (Figs. 2a and 3a).

Each seminiferous tubule was surrounded by basement membrane and myoid cells with flat nuclei. Seminiferous tubules were lined with stratified spermatogenic cells and Sertoli cells. Spermatogenic cells included: spermatogonia, primary spermatocytes, secondary spermatocytes, spermatids, and spermatozoa. Spermatogonia were resting on a basement membrane. Spermatocytes are the largest spermatogenic cells. Two forms of spermatids were seen; round (early spermatids) and elongated (late spermatids). Mature spermatozoa were seen filling the lumen of the seminiferous tubules. Sertoli cells appeared near the basement membrane (Figs. 2a and 3a).

After administration of the toxic dose of AuNPs for 28 days, marked reduction of stratified germinal epithelium lining seminiferous tubules appeared. Most of spermatogenic cells were replaced by vacuoles. The lumens of most of seminiferous tubules were devoid of mature spermatozoa. Sertoli cells and Leydig cells showed marked degeneration. Blood vascular walls appeared thickened. Interstitial inflammation and edema were detected (Figs. 2b and 3b). Compared to the control group, the test group showed statistically significant disruption of seminiferous tubules, detachment of spermatogenic cells, interstitial inflammation and edema, congestion of vessels, degeneration of Sertoli cells and degeneration of Leydig cells (*p1* < 0.001 for all) (Table 7).

**Table 7:** Comparison of testicular histopathological score results among all studied groups of rats (n = 60)

Groups (n = 15 for each)	Control group	Test group	Withdrawal group I	Withdrawal group II	Test of significance	Paired comparison
Disruption of seminiferous tubules Median (min- max)	0 (0–0)	2.25 (1–3)	0.85 (0.2–1.7)	0.005 (0–0.04)	KW *p* < 0.001*	*p1* < 0.001*
						*p2* < 0.001*
						*p3* = 0.063
						*p4* = 0.001*
						*p5* < 0.001*
						*p6* = 0.001*
Detachment of spermatogenic cells Median (min- max)	0 (0–0)	2.85 (1–3)	0.95 (0.5–1.7)	0 (0–0.3)	KW *p* < 0.001*	*p1* < 0.001*
						*p2* < 0.001*
						*p3* = 0.068
						*p4* = 0.002*
						*p5* < 0.001*
						*p6* < 0.001*
Inflammation Median (min- max)	0 (0–0)	1.35 (1–3)	0 (0–0.5)	0 (0–0.8)	KW *p* < 0.001*	*p1* < 0.001*
						*p2* < 0.001*
						*p3* = 0.147
						*p4* = 0.001*
						*p5* < 0.001*
						*p6* = 0.691
Edema of interstitium Median (min- max)	0 (0–0)	2.65 (1–3)	0.75 (0.3–1.3)	0 (0–0.1)	KW *p* < 0.001*	*p1* < 0.001*
						*p2* = 0.001*
						*p3* = 0.317
						*p4* < 0.001*
						*p5* < 0.001*
						*p6* = 0.013*
Congestion of vessels Median (min- max)	0 (0–0)	1.75 (1–3)	0.15 (0–0.5)	0 (0–0.1)	KW *p* < 0.001*	*p1* < 0.001*
						*p2* = 0.005*
						*p3* = 0.063
						*p4* < 0.001*
						*p5* < 0.001
						*p6* = 0.481
Sertoli cells degeneration Median (min- max)	0 (0–0)	2.75 (1–3)	0.75 (0.4–1.5)	0 (0–0.2)	KW *p* < 0.001*	*p1* < 0.001*
						*p2* < 0.001*
						*p3* = 0.147
						*p4* < 0.001*
						*p5* < 0.001*
						*p6* = 0.001*
Leydig cells degeneration Median (min- max)	0 (0–0)	2.25 (1–3)	0.15 (0–0.5)	0 (0–0.8)	KW *p* < 0.001*	*p1* < 0.001*
						*p2* < 0.001*
						*p3* = 0.147
						*p4* < 0.001*
						*p5* < 0.001*
						*p6* = 0.008*

*n* number, *min* minimum, *max* maximum, *p* comparison among all studied groups, *p1* comparison between control group and test group, *p2* comparison between control group and withdrawal group I, *p3* comparison between control group and withdrawal group II, *p4* comparison between test group and withdrawal group I, *p5* comparison between test group and withdrawal group II, *p6* comparison between withdrawal groups I and II, *KW* Kruskal Wallis test. *p is significant

After withdrawal of the toxic dose of AuNPs for 30 days and continuing on standard diet and water only, some seminiferous tubules showed preservation of the normal stratified germinal epithelium. Meanwhile, other tubules were lined by vacuolated and degenerated cells (Figs. 2c and 3c).Compared to the test group, withdrawal group I showed statistically significant decrease in disruption of seminiferous tubules, detachment of spermatogenic cells, interstitial inflammation and edema, congestion of vessels and degeneration of Sertoli cells and Leydig cells (*p4* = 0.001, = 0.002, = 0.001, < 0.001, < 0.001, < 0.001 and < 0.001 respectively) (Table 7).

After withdrawal of the toxic dose of AuNPs for 60 days and continuing on standard diet and water only, near normal testicular architecture was observed (Figs. 2d and 3d). There was statistically significant decrease in disruption of seminiferous tubules, detachment of spermatogenic cells, interstitial inflammation and edema, congestion of vessels and degeneration of Sertoli cells and Leydig cells compared to that of the test group (*p5* < 0.001 for all) (Table 7).

#### Digital morphometric study (computer assisted digital image analysis)

The test group showed a statistically significant increase in the thicknesses of tunica albuginea and blood vascular walls and the mean diameter of seminiferous tubules and a statistically significant decrease in the thickness of the epithelial lining of the tubule compared to the control group (*p1* < 0.001 for all).

On the other hand, withdrawal groups I and II showed statistically significant decreases in the thicknesses of tunica albuginea and blood vascular walls and a statistically significant increase in the thickness of the epithelial lining of the tubule compared to the test group. In addition, withdrawal group II showed a statistically significant decrease in the mean diameter of seminiferous tubules compared to the test group (Table 8).

**Table 8:** Comparisons of digital image analysis results among all studied groups of rats (n = 60)

Groups (n = 15 for each)	Thickness of tunica albuginea (μm) Mean ± SD	Thickness of the blood vascular walls (μm) Mean ± SD	Thickness of the epithelial lining of the tubule (μm) Mean ± SD	Diameters of seminiferous tubules (μm) Mean ± SD
Control group	8.46 ± 1.41	5.98 ± 2.91	41.71 ± 9.38	140.83 ± 23.16
Test group	40.08 ± 8.47	18.36 ± 8.48	15.56 ± 5.39	234.32 ± 87.45
Withdrawal group I	18.57 ± 4.28	8.66 ± 1.65	26.43 ± 5.33	197.67 ± 67.05
Withdrawal group II	10.27 ± 1.41	7.62 ± 1.75	37.39 ± 7.21	169.09 ± 17.46
Test of significance	F = 161.08	F = 26.02	F = 50.18	F = 8.87
	*p* < 0.001*	*p* < 0.001*	*p* < 0.001*	*p* < 0.001*
Paired comparison	*p1* < 0.001*	*p1* < 0.001*	*p1* < 0.001*	*p1* < 0.001*
	*p2* < 0.001*	*p2* = 0.09	*p2* < 0.001*	*p2* = 0.004*
	*p3* = 0.264	*p3* = 0.293	*p3* = 0.147	*p3* = 0.141
	*p4* = 0.001*	*p4* = 0.001*	*p4* = 0.001*	*p4* = 0.058
	*p5* < 0.001*	*p5* < 0.001*	*p5* < 0.001*	*p5* = 0.001*
	*p6* < 0.001*	*p6* = 0.504	*p6* = 0.691	*p6* = 0.137

*n* number, *μm* micrometer, *SD* standard deviation, *p* comparison among all studied groups, *p1* comparison between control group and test group, *p2* comparison between control group and withdrawal group I, *p3* comparison between control group and withdrawal group II, *p4* comparison between test group and withdrawal group I, *p5* comparison between test group and withdrawal group II, *p6* comparison between withdrawal groups I and II, *F* One Way ANOVA test. *p is significant

### Electron microscopic results

**Fig. 4:**
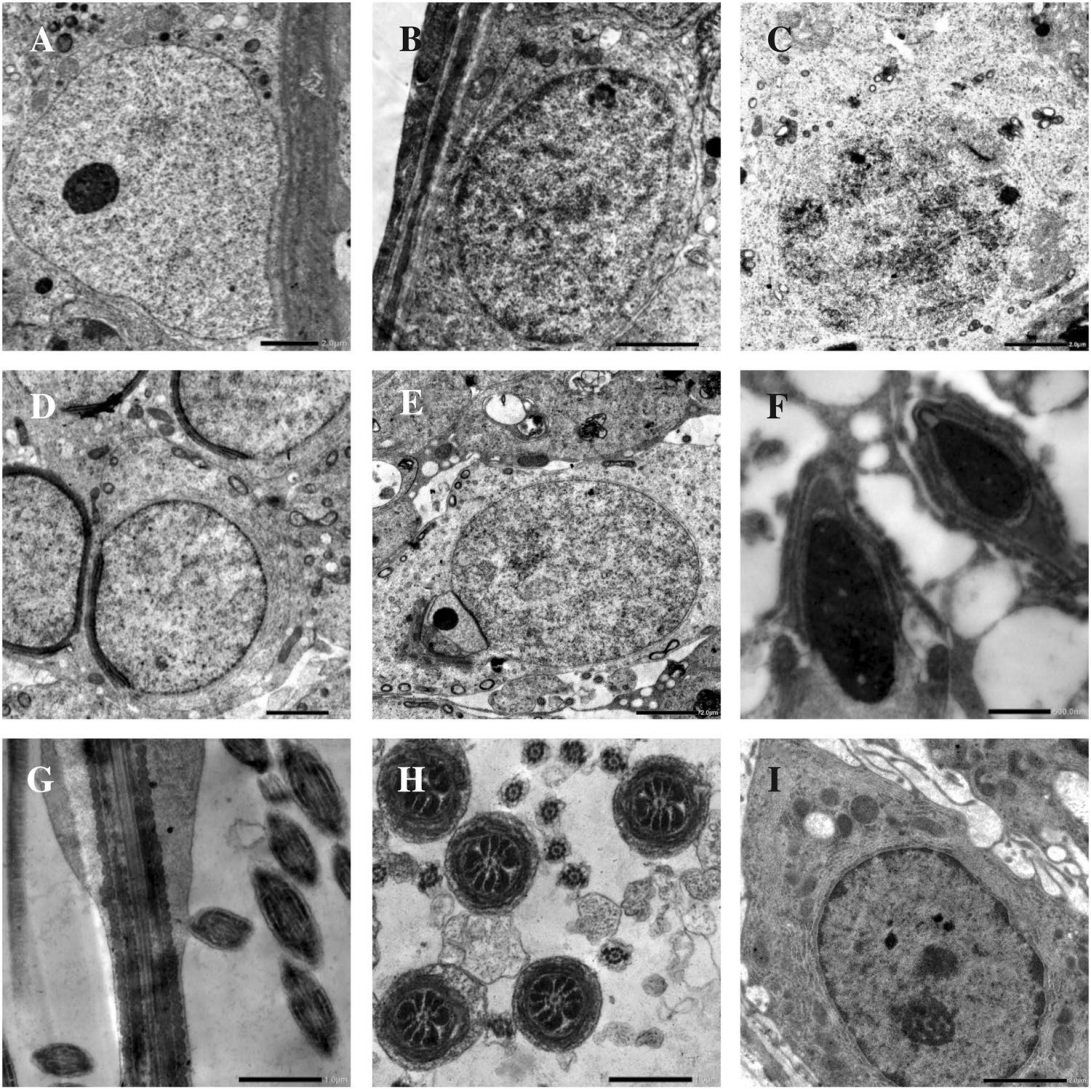
Electron micrographs of the testicular cells of the control group showing Sertoli cell resting on basal lamina (**a**), spermatogonium resting on basal lamina (**b**), primary spermatocytes (**c**), round spermatids (**d** and **e**), head of mature sperm (**f**), longitudinal section of mature sperm tail (**g**), transverse section of mature sperm tail (**h**) and normal interstitial Leydig cell (i). Scale bar: 2 μm (**a**, **b**, **c**, **d**, **e** and **i**), 1 μm (**g** and **h**) and 500 nm (**f**). Number of observations: 5 images per each testicular cell type

**Fig. 5:**
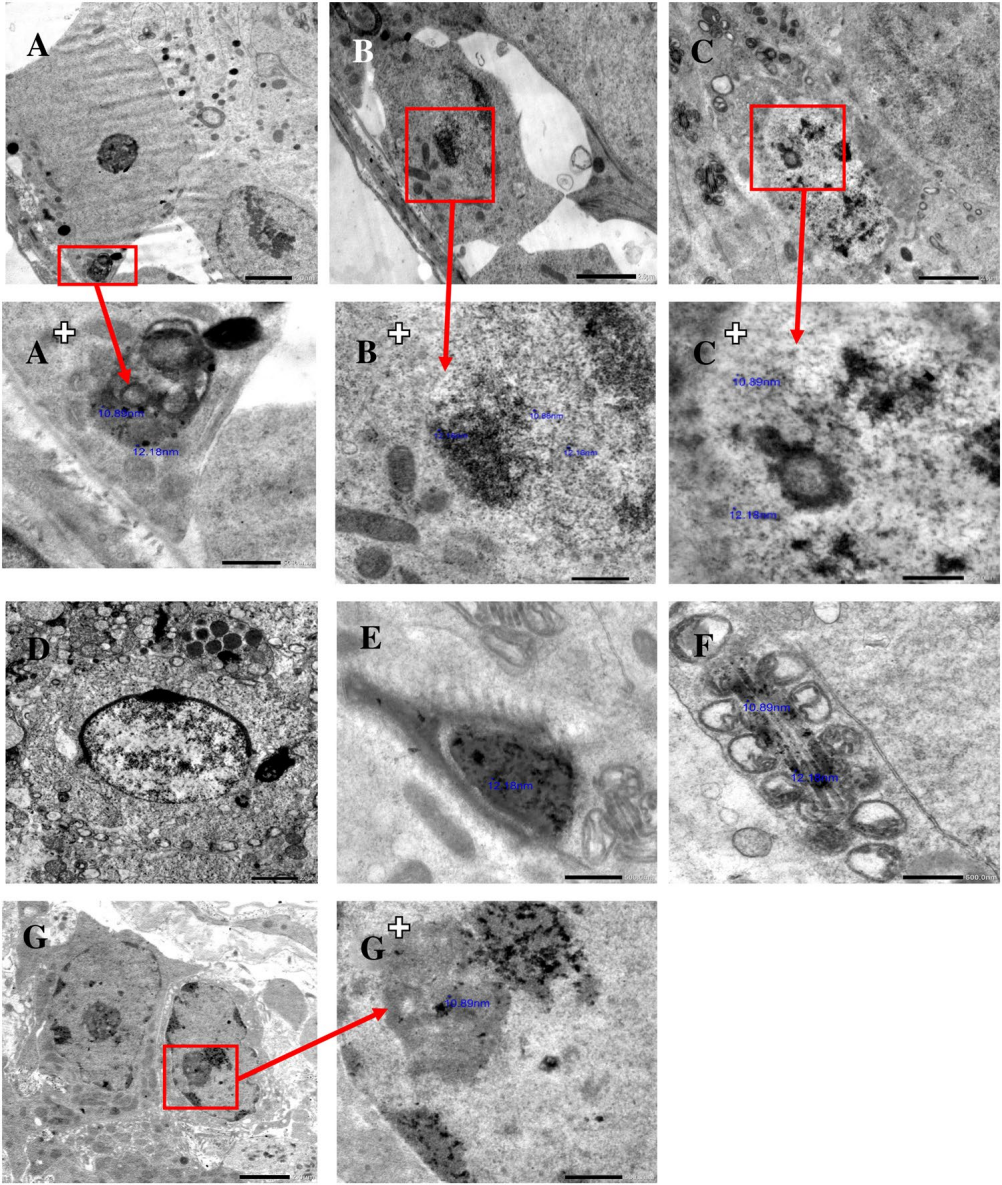
Electron micrographs of the testicular cells of the test group revealing seminiferous tubules bounded by thick wavy basal lamina (**a** and **b**). a + , b + , c + and g + are more magnified photos of a, b, c and g photos to show AuNPs accumulation inside Sertoli cell, spermatogonium, primary spermatocyte, mature sperm head and tail and Leydig cell (a + , b + , c + , e, f and g + respectively). Wide intercellular spaces are shown between Sertoli cell, spermatogonium and their adjacent cells (**a** and **b**). Spermatogonium and primary spermatocyte show disrupted nuclear membrane (**b** and **c** respectively). Round spermatid shows peripherally arranged vaculated mitochondria (**d**). Leydig cells show shrunken nucleus and excess lipid droplets (**g**). Cytoplasmic vacuolization and mitochondrial degeneration are present in almost all testicular cells. Scale bar: 2 μm (a, b, c, d and g) and 500 nm (a^+^, b^+^, c^+^, e, f and g^+^). Number of observations: 5 images per each testicular cell type

**Fig. 6:**
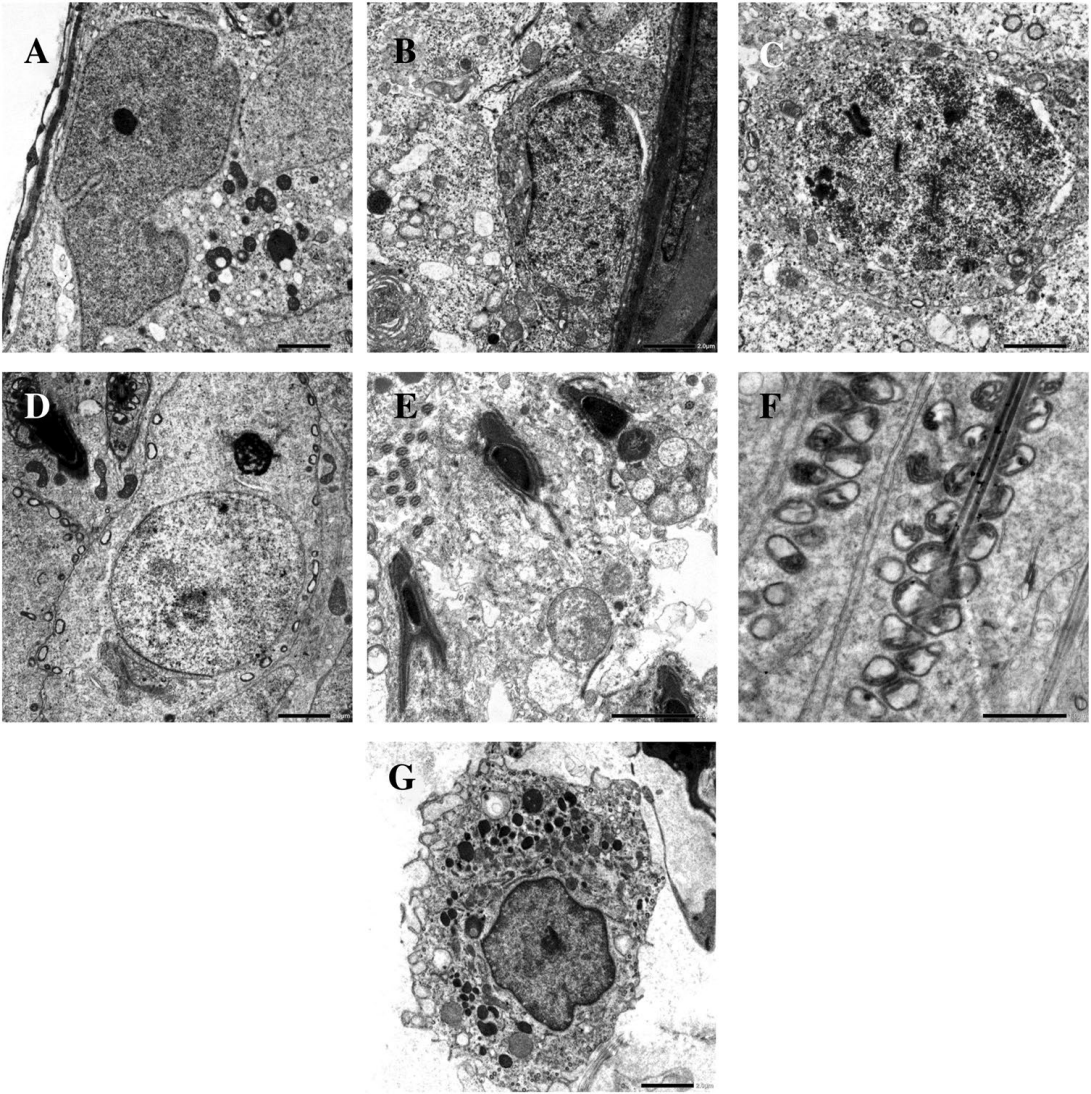
Electron micrographs of the testicular cells of withdrawal group I showing Sertoli cell with normal indented nuclear membrane, vacuolization and mitochondrial degeneration (**a**), spermatogonium and primary spermatocyte with irregular nuclear membranes, perinuclear spaces, vacuolization and mitochondrial degeneration (**b** and **c** respectively), normal round spermatid (**d**), heads and tails of mature sperms with vaculated mitochondria (**e** and **f** respectively). Leydig cell with degenerated mitochondria and excess lipid droplets (**g**). Scale bar: 2 μm (**a**, **b**, **c**, **d**, **e** and **g**) and 1 μm (**f**). Number of observations: 5 images per each testicular cell type

**Fig. 7:**
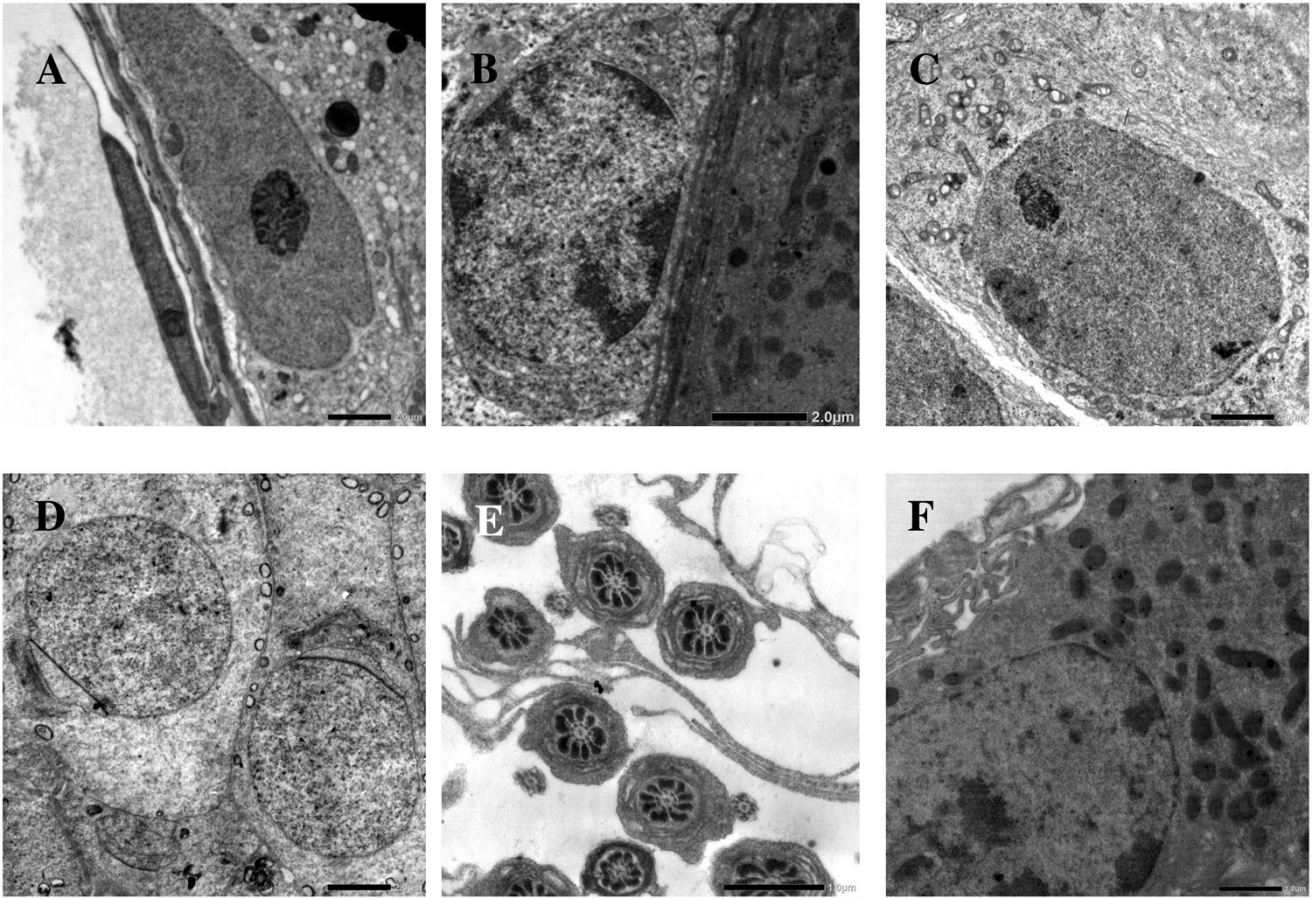
Electron micrographs of the testicular cells of withdrawal group II showing near normal thickness basal lamina (**a** and **b**), Sertoli cell with normal indented nuclei and prominent nucleoli, its cytoplasm contains mitochondria and small vacuoles (**a**), normal spermatogonium (**b**), primary spermatocyte with few vaculated mitochondria (**c**), normal round spermatids (**d**), normal transverse sections of mature sperm tails (**e**), normal Leydig cell (f). Scale bar: 2 μm (**a**, **b**, **c** and **d**) and 1 μm (**e** and **f**). Number of observations: 5 images per each testicular cell type

The control group showed normal testicular ultrastructural architecture (Fig. 4). In comparison, the test group's testicular cells showed vacuolization, mitochondrial degeneration, nuclear membrane disruption, and wide intercellular spaces associated with AuNPs accumulation inside Sertoli cells, spermatognia, spermatocytes, mature sperm heads and tails, and Leydig cells (Fig. 5). Furthermore, some testicular cells of withdrawal group I show nuclear membrane irregularity, perinuclear spacing, vacuolization, and mitochondrial degeneration, while other testicular cells are normal (Fig. 6). Moreover, most testicular cells in withdrawal group II are normal, with few small vacuoles and few degenerated mitochondria (Fig. 7).

## Discussion

Nanoparticles are currently utilized in every branch of science and in commercial applications to make products cleaner, lighter, stronger, more precise, more efficient, and more aesthetic [5]. Among them, AuNPs are highly remarkable with their unique functional properties and easy synthesis, allowing them to be used in a wide range of medical applications, including biosensing, photothermal therapy, photodynamic therapy, radiotherapy, X-ray imaging, computed tomography, and gene and drug delivery [14]. Due to the fact that NPS may negatively affect male reproductive organs, spermatogenesis, and hormone levels, reproductive toxicity caused by NP exposure is regarded as an essential topic to be researched in general toxicology [16].

This study aimed at studying the toxic effects of AuNPs on the reproductive system of adult male Albino rats and assessing their reversibility after 30 and 60 days of withdrawal. To the best of the authors' knowledge, there are no available studies investigating the reversibility of AuNPs-induced reproductive toxicity.

In this experimental study, sixty adult male Albino rats were divided into four groups (fifteen rats each): the control group, the test group, withdrawal group I, and withdrawal group II. Control group rats received deionized water daily through the intraperitoneal route for 28 days. Test group and withdrawal groups I and II rats received 570 μg/kg of AuNPs (13 ± 4 nm) daily through the intraperitoneal route for 28 days. Then, withdrawal groups I and II continued for another thirty and sixty days, respectively, with free access to tap water and food to assess the recovery.

Several researchers have already utilized the animal model used in this study to evaluate the AuNPs induced reproductive toxicity [10, 13, 35]. Regarding the AuNPs dose used in the current study, Zhang et al. [41] have used an equivalent dose in mice, and this dose was calculated in rats according to Nair and Jacob [27] conversion tables. The same dose was also used in rats by Velikorodnaya et al. [35] to evaluate AuNP-induced reproductive toxicity. The intraperitoneal route was preferred due to the dense blood vessels and lymph in the peritoneum, which allow good and rapid drug absorption [41].

In the present study, there was statistically significant decrease in final body weights in animals of the test group compared to animals of the control group. Zhang et al. [41] obtained a similar result following daily IP injection of mice with AuNPs (13.5 nm) for 28 days. The metabolic effects of AuNPs, which have been reported by Chen et al. [6], could be the cause of this outcome. They found that IP injection of AuNPs (20–30 nm) into mice improved lipid and glucose metabolism, decreased fat mass, and aided in weight loss. However, compared to the test group, withdrawal groups I and II showed statistically non-significant and statistically significant increases in final body weights, respectively. When the final body weights of both withdrawal groups were compared, withdrawal group II showed a statistically significant increase. This could be attributed to the gradual recovery from AuNPs-induced toxicity after AuNPs clearance from the body.

Furthermore, the test group showed a statistically significant decrease in absolute testicular weights compared to the control group. Meanwhile, withdrawal groups I and II showed statistically non-significant and statistically significant increases in absolute testicular weights compared to the test group, respectively. The toxic effects of NPs on germ cell mass could be the cause of the test group's decreased absolute testicular weight [11]. While, its increase in withdrawal groups I and II indicated gradual regeneration of germ cells and gradual recovery from AuNPs induced spermatogenic defects.

Regarding serum testosterone hormone levels, they were statistically decreased in the test group compared to the control group. This result was in agreement with Behnammorshedi et al. [4] who observed that after daily IP injection of different doses of AuNPs for ten days in rats, the mean testosterone level decreased with increasing AuNPs dose. Similarly, Liu et al. [21] suggested that after daily intravenous injection of AuNPs (5–10 nm) for 14 days in mice, the testosterone production in Leydig cells reduced due to down regulating of the expression of 17α hydroxylase enzyme, which has crucial importance in androgen synthesis and degeneration of Leydig cells which are responsible for testosterone production.

Meanwhile, withdrawal groups I and II showed statistically significant increases in serum testosterone hormone levels compared to the test group. This result may be attributed to regaining of Leydig cells mitochondrial secretory activity after resolving their degeneration.

As regards semen analysis, the test group displayed statistically significant decreases in sperm count and percentage of motile sperms and a statistically significant increase in the percentage of sperms with abnormal morphology compared to the control group. These results were in accordance with Wiwanitkit et al. [36], who reported the effect of mixing AuNPs (9 nm) with a fresh semen sample from a healthy human male. They observed that after 15 min of exposure to AuNPs, the motility was lost in 25% of the sperms, and some human sperms were clumped and fragmented with an accumulation of AuNPs in the sperm tails and heads. Another study conducted by Taylor et al. [32] on bovine spermatozoa reported detrimental effects on sperm motility, morphology, and fertilizing capability after mixing with AuNPs (10.8 nm in average).

As well, Nazari et al. [28] reported a significant decrease in sperm motility and an increased number of abnormal spermatozoa after repeated IP injection of AuNPs (10–30 nm) in mice. In addition, Liu et al. [21] reported sperm malformations (including small heads, large heads, double heads, double tails, and coiled tails) after daily intravenous injection of AuNPs (5–10 nm) for 14 days in mice.

These detrimental effects of AuNPs on sperm quantity and quality were explained by the induction of reactive oxygen species (ROS), resulting in oxidative stress and mitochondrial damage with subsequent metabolic dysfunction [11].

Furthermore, withdrawal groups I and II showed statistically significant increases in sperm count and percentage of motile sperms and decrease in the percentage of sperms with abnormal morphology compared to the test group. This result reflected the gradual reversibility of AuNPs-induced damage to the epididymal sperms.

Concerning light microscopic results, the test group showed statistically significant decreases in JTBS for spermatogenesis and the mean thickness of the epithelial lining of the tubules compared to the control group. The negative effect of AuNPs on spermatogenesis in the current study may be attributed to their ability to produce ROS, which leads to formation of oxidative stress and disruption of cellular metabolism associated with inducement or exacerbation of the NPs-related inflammatory response [38], as well as induction of oxidative DNA damage that leads to cell cycle arrest and cytotoxic effects on male germ cells. This explains why sperms and spermatids were rarely seen in the test group [11].

Regarding other light microscopic results, the test group showed statistically significant disruption of seminiferous tubules, detachment of spermatogenic cells, interstitial inflammation and edema, congestion of vessels, and degeneration of Sertoli and Leydig cells compared to the control group. The mean thicknesses of tunica albuginea and blood vascular walls and the mean diameter of the seminiferous tubules showed statistically significant increases compared to the control group.

These toxic testicular histopathological changes could be explained by the intracellular leaching of gold ions from AuNPs and their effects on the surrounding biomacromolecules. Consequently, these ions strongly inhibit mitochondrial membrane depolarization and/or inactivation of mitochondrial enzymes, rendering direct or indirect mitochondrial damage, leading to alteration of cellular redox balance and promoting cell necrosis or apoptosis [34].

Furthermore, testicular interstitial inflammation and edema generated in the current study could be explained by AuNPs activation of inflammatory mediators' synthesis by disturbing the normal mechanisms of cell metabolism [17]. Additionally, congestion of blood vessels and interstitial edema could be attributed to the induction of nitric oxide production, which is an endothelial relaxing factor [23].

Moreover, the thickening of tunica albuginea, basal lamina, and blood vascular walls observed in the test group of the current study can be attributed to increased production of glycosaminoglycans and proteoglycans, a mechanism that is considered a defense reaction against the damaging activity of the probably induced ROS [3].

Although the mean thickness of the epithelial lining of seminiferous tubules was decreased in the test group of the current study, their mean diameters were increased. This could be explained as a result of the detachment of spermatogenic cells into the lumen, leading to blocking of the efferent ducts with subsequent impairment of seminiferous tubule fluid passage from the testis to the epididymis, resulting in increased seminiferous tubule diameter [25].

Regarding the electron microscopic results of the test group, most of the intratubular and interstitial testicular changes could be explained by lipid peroxidation of the cell membranes and organelles. It also destroys the structure of the spermatozoal lipid matrix, which can be associated with loss or affect sperm motility [7].

Cytoplasmic vacuolization of testicular cells in the test group of the current study might have arisen from lysosomal membrane damage induced by ROS with subsequent release of lysosomal hydrolases into the cytosol, uncontrolled extra lysosomal proteolysis, and tissue destruction [15]. The clear vacuoles within the cytoplasm might represent distended and pinched-off segments of the endoplasmic reticulum. The cellular swelling might occur as a result of failure of energy-dependent sodium–potassium ion pumps in the plasma membrane, leading to intracellular accumulation of sodium and progressive changes in osmolarity with consequent entry of water into the cells. This pattern of injury could be referred to as hydropic change [19].

The accumulation of AuNPs in Sertoli cells, spermatogenic cells, and Leydig cells in the current study was strong evidence of their ability to cross the blood testis barrier (BTB). AuNPs accumulation in Sertoli cells and its associated Sertoli cell degeneration would have altered the structural and functional integrity of testicular tissues, with subsequent disruption of the Sertoli-germ cell interaction leading to the detachment of spermatogenic cells from the seminiferous epithelium. Moreover, Sertoli cell degeneration would have impaired the production of growth factors and nutrients, which would have a harmful impact on the normal maturation of spermatogenic cells at various stages [33]. In addition, AuNPs accumulation inside the interstitial Leydig cells and its subsequent degeneration explain the decrease in testosterone levels and impaired sperm production and maturation of the test group rats [21].

Mohamed et al. [26] also noted the presence of intercellular gaps between the spermatogenic cells as found in the test group of the current study. They attributed this to the disruption of tight junctions in BTB upon exposure to the ROS, leading to the entry of excess water and toxic agents between the spermatogenic cells and widening of intercellular spaces.

Both light and electron microscopic results of the test group came in accordance with Gupta et al. [10], who stated that after oral exposure of mice to AuNPs (15 nm) for 90 days, there was considerable accumulation of AuNPs in the testes, degeneration of testicular tissues, detachment of germinal epithelium from the basement membrane, and a reduction in the population of germ cells. Also, these results came in agreement with Liu et al. [21], who performed combined in vitro and in vivo studies on Leydig cells and mice and recognized that after AuNPs (5 nm) internalization into Leydig cells lysosomes, they induced the formation of autophagosomes, increased the production of ROS, and arrested the cell cycle in S phase, resulting in concentration-dependent cytotoxicity and DNA damage with a significant reduction of testosterone production. Additionally, after daily intravenous injections of AuNPs for 14 days in mice, they accumulated and were retained in the testes in a dose-dependent manner.

However, the results of the current study were in disagreement with Leclerc et al. [20], who reported that after daily intramuscular injection of AuNPs (70 nm) for 45 days, there were neither testicular histopathological toxicity signs nor AuNPs testicular accumulation. This inconsistency of results could be due to different sizes of AuNPs and different routes of their administration.

Both light and electron microscopic results of withdrawal groups I and II of the present study reflected the gradual reversibility of AuNPs-induced damage to the testicular tissues. Similar reversible effects for the damage of the testicular tissues were detected by Bai et al. [2], who used carbon nanotubes, Ren et al. [30], who used silica nanoparticles,and Nirmal et al. [29], who used graphene oxide nanoparticles.

In this study, the reversibility of AuNPs-induced male reproductive toxicity may be attributed to: (A) exocytosis of NPs as suggested by Sakhtianchi et al. [31], (B) clearance of AuNPs from a variety of body organs as investigated by Han et al. [12], (C) deoxyribonucleic acid repair process as detected by Xu et al. [37], and (D) subsidence of AuNPs-induced oxidative stress and lipid peroxidation as concluded from other nanoparticle reversibility studies [2, 30].

## Recommendations

Health programs should be conducted to provide the public with information about AuNPs-containing products and their male reproductive toxic effects associated with limiting the use of these products to the necessary ones only. In addition, precautions should be taken when dealing with AuNPs-containing products, especially in terms of occupational work, by wearing gloves and protective clothes. Patients on AuNPs treatment should be reassured that male reproductive toxicity is reversible.

### Supplementary Information

Below is the link to the electronic supplementary material.

Supplementary file1 (XLSX 19 KB)

## Data Availability

All datasets on which the conclusions of the manuscript rely are available.
